# Amelioration of cyclophosphamide-induced myelosuppression during treatment to rats with breast cancer through low-intensity pulsed ultrasound

**DOI:** 10.1042/BSR20201350

**Published:** 2020-09-24

**Authors:** Wei Wang, Dong Luo, Junlin Chen, Jinyun Chen, Yi Xia, Wenzhi Chen, Yan Wang

**Affiliations:** State Key Laboratory of Ultrasound in Medicine and Engineering, College of Biomedical Engineering, Chongqing Key Laboratory of Biomedical Engineering, Chongqing Medical University, Chongqing, China

**Keywords:** Breast cancer, chemotherapy, hematopoiesis, inflammation, low-intensity pulsed ultrasound, myelosuppression

## Abstract

To investigate the alleviating effects of low-intensity pulsed ultrasound (LIPUS) on myelosuppression of Sprague–Dawley rats with breast cancer induced by cyclophosphamide (CTX). Breast cancer in rats was triggered by intragastric gavage with 7,12-dimethylbenz[a]anthracene (150 mg/kg). Then, the rats with breast cancer were randomly allocated to the LIPUS group (*n*=50) and the control group (*n*=50). The LIPUS group was injected intraperitoneally with CTX (50 mg/kg) for 4 consecutive days and underwent LIPUS treatment at femoral metaphysis 20 min per day from the first day of injection for 7 consecutive days. The control group was injected with CTX (50 mg/kg) and treated with LIPUS without energy output. Blood, enzyme-linked immunosorbent assay (ELISA), real-time quantitative polymerase chain reaction, Hematoxylin and Eosin (H&E) staining, and scanning electron microscopy were applied to detect the changes. The results indicated that LIPUS significantly promoted the proliferation of bone marrow nucleated cells, white blood cells (WBCs), IgA, IgG, and IgM in the peripheral blood (*P*<0.05) without the damage to liver and kidney function simultaneously. The mechanisms may result from the LIPUS alleviation effect on bone marrow hematopoietic function through regulating cytokines such as LIPUS can increase the expression of granulocyte colony-stimulating factor (G-CSF), stem cell factor, transforming growth factor-β, and intercellular cell adhesion molecule-1, meanwhile LIPUS will decrease the expression of interleukin-6, tumor necrosis factor-α, and vascular cell adhesion molecule-1. LIPUS has potential to be a new adjuvant therapy method in clinic for ameliorating chemotherapy-induced myelosuppression.

## Introduction

Breast cancer is the most frequently diagnosed cancer in women worldwide and the leading cause of cancer-related deaths among women in less-developed countries [1,2]. Chemotherapy is one of the main effective systemic treatments for breast cancer, in which cyclophosphamide (CTX) is widely used in the early stages [3]. However, during treatment of breast cancer leukopenia, and neutropenia, is the most common which is triggered by cytotoxic anticancer drugs having dose-limiting toxicity [4,5]. Severe leukopenia and neutropenia often result in treatment delay or dose reduction and discontinuation. In addition, patients with neutrophil have more potential to be infected or have any other adverse case to make the quality of life and subsequent treatment worse [6]. Granulocyte colony-stimulating factors (G-CSFs) known as a drug used to treat neutropenia, such as filgrastim, lenograstim, and pegfilgrastim are widely used in clinical practice to reduce the risk of chemotherapy-induced neutropenia [7–10]. However, G-CSF will also cause side effects including mild and transient reactions of headache, bone pain, myalgia, fever, flushing, rash, and so on. It will even result in some severe but rare side effects including respiratory, hematologic, and cutaneous reactions [11–17]. Therefore, a new method with safer, more effective, and less harmful to treat leukopenia caused by chemotherapeutic drugs should be sought.

Low-intensity pulsed ultrasound (LIPUS) is proven to be an inexpensive, safe, and non-invasive treatment method [18–21]. The latest findings indicated that LIPUS treatment could significantly promote the proliferation of bone marrow nucleated cells and white blood cells (WBCs). It also promoted the differentiation of hematopoietic stem cells into granulocytes and lymphocytes [22]. Previous reports about the therapeutic effects of LIPUS on leukopenia induced by chemotherapy drugs just worked on normal animals which did not have cancer [22,23]. However, it is not clear whether LIPUS can treat CTX-induced leukopenia in animals bearing cancer. In order to be closer to the actual situation of clinical application, the present study was designed to explore the effects and mechanisms of LIPUS on ameliorating CTX-induced myelosuppression in rats with breast cancer. Combined with the previous studies, it will provide experimental support for the clinical application of LIPUS in future.

## Materials and methods

### Experimental animals

SD rats (*n*=150, female, 6–8 weeks, 160–180 g) were provided by the Experimental Animal Center of Chongqing Medical University (Production License No. SCXK 2017-0023, Chongqing, China). These rats were placed in animal houses with standard conditions (temperature 23 ± 5°C, light time 12 h/day, humidity 45 ± 5%) with free access to standard water and food. All experiments were applied following the Guide for the Care and Use of Laboratory Animals issued by the Ministry of Science and Technology, China. The protocol of experiment was confirmed by the Ethics Committee of the Chongqing Medical University. All animal experiments were conducted at the Experimental Animal Center, Chongqing Medical University, China.

### Breast cancer caused by 7,12-dimethylbenz[a]anthracene

The breast cancer of SD rats (*n*=140) was induced by using 150 mg/kg of 7,12-dimethylbenz[a]anthracene (Sigma–Aldrich, St. Louis, MO, U.S.A.) for gavage [24]. Normal rats (*n*=10) were left untreated. The general conditions of rats including weight, eating, drinking, activity, and mental state were observed daily. The diameter of the tumor was observed and measured every week. The breast tissues were applied to a pathological examination to check whether the model is successful.

### Chemotherapy with CTX

Rats that died in the process of modeling and those with very small tumor volumes were excluded from the group, and finally the remaining 100 rats were entered into the groups. The rats with breast cancer were randomly divided into two groups: control group (*n*=50) and LIPUS group (*n*=50). The two groups were given an intraperitoneal injection of CTX (50 mg/kg) (Shanxi Pude Pharmaceutical Co., Ltd., Datong, Shanxi, China) for 4 days.

### LIPUS treatment

The LIPUS device was provided from Chongqing Haifu Medical Technology Co., Ltd, Chongqing, China. In the LIPUS group, 20-min ultrasound treatment was performed at the femoral metaphysis from days 1 to 7. The rats of LIPUS group (*n*=50) were intraperitoneally anesthetized with pentobarbital sodium (35 mg/kg body weight) and then adopted the LIPUS treatment. The right femoral metaphysis of rats was selected and the skin of the femur parts of the rats was prepared. The ultrasound transducer was applied to the femoral metaphyseal skin of the rats. An ultrasound coupling agent (Tianjin Chengxin Medical Auxiliary Material Factory, Tianjin, China) was applied between the transducer and the skin, and the transducer was not moved during the treatment. Ultrasound parameters: intensity 200 mW/cm^2^, frequency 0.3 MHz, repetition frequency 1 kHz, duty cycle 20%. The control group received sham treatment.

### Peripheral blood cell counts analysis

To find the change in WBC, neutrophil, lymphocyte in peripheral blood, which were detected using an automatic blood cell count analyzer (Bowlinman Sunshine Co., Ltd, Beijing, China). The rats of each group (*n*=10) were intraperitoneally anesthetized with pentobarbital sodium (35 mg/kg body weight) and then the blood (0.5 ml) was collected from retro-orbital venous plexus of each rat using vacuum blood collection tube (Yuli Medical Equipment Co., Ltd, Jiangsu, China) containing ethylenediaminetetraacetic acid (EDTA) anticoagulant. Peripheral blood samples were taken on days 0 (before chemotherapy), 4, 7, and 14. Normal rats (*n*=10) peripheral blood was collected in the same way.

### Function detection of liver and kidney

To evaluate the effect of 7,12-dimethylbenz[a]anthracene, CTX, and LIPUS on liver and kidney, alanine transaminase (ALT), total bilirubin (TBIL), blood urea nitrogen (BUN), and creatinine (CRE) were detected by Automatic Biochemistry Analyzer (Sysmex, Japan). Rats in each group (*n*=10) were anesthetized in the same way and the blood (1.5 ml) was collected from retro-orbital venous plexus of each rat using vacuum blood collection tube (Yuli Medical Equipment Co., Ltd, Jiangsu, China). The blood samples were taken on days 0 and 7.

### Enzyme-linked immunosorbent assay

The blood (2 ml) was collected from each group (*n*=10) by the aforementioned method to assess change of cytokines on days 0, 4, 7, and 14. Blood was taken from normal rats (*n*=10) in the same way. The serum was isolated from blood by centrifugation at 3000 rpm for 15 min. The IgA, IgG, IgM, G-CSF, IL-6, and TNF-α levels were checked using enzyme-linked immunosorbent assay (ELISA) kits (Hushang, Shanghai, China) according to the manufacturer’s protocols. The samples were added to the enzymatic standard holes prepackaged with relevant monoclonal antibodies. After warm incubation, the antibodies labeled with biotin were added and combined with streptomycin–HRP to form an immune complex, which was then incubated and washed to remove unbound enzymes. The color development reagents were added to show blue color, which was converted into the final yellow color under the action of acid.

### Reverse transcription-quantitative polymerase chain reaction

The bone marrow was taken from the femur of each rat on day 7 to detect the gene expression. Rats in each group (*n*=10) were over-anesthetized to be euthanized. Following adequate anesthesia with pentobarbital sodium (170 mg/kg body weight), the femur was separated from the body, temporarily soaked in 75% ethanol, and rinsed three times in phosphate buffer solution (PBS) under aseptic conditions. The epiphysis of each bone was removed using the blade. PBS was used to rinse the marrow cavity through the syringe needle several times. Then, the marrow cell suspension was centrifuged at 1000 rpm for 10 min, and the sediment was stored in the refrigerator at −80°C for reverse transcription-quantitative polymerase chain reaction (RT-qPCR) detection and analysis. The total mRNA in the bone marrow was extracted by the TRIzol method, and the RNA concentration was detected by ultraviolet spectrophotometry. The RNA (1 μg) extracted from each sample was reverse-transcribed into cDNA using a ReverTra Ace-a cDNA kit (TOYOBO Biotechnology Co., Ltd. Tokyo, Japan). The nucleotide sequences of the forward and reverse primers used for PCR are shown in Table 1. The experiment was performed using the CFX96 RT-qPCR System (Bio-Rad, CA, U.S.A.). The results of the gene expression of mRNA in each group were analyzed by the comparative *C*_t_ method.

**Table 1:** Primers used for RT-qPCR

Genes	Forward (5′–3′)	Reverse (5′–3′)
*SCF*	5′-TGCAATATGAAGCCCCAAGACAC-3′	5′-CTCATGGTGCCCTCCTGCTACTT-3′
*TGF-β*	5′-GCACCGGAGAGCCCTGGATAC-3′	5′-ATCCACTTCCAACCCAGGTCCT-3′
*ICAM-1*	5′-CCACCCGGCTCCACCTCAAAG-3′	5′-CTGCGCTGGGAGGGGTGC-3′
*VCAM-1*	5′-CCCCTCCACAAACCAAGCTATGC-3′	5′-TCCGGTCTCTTCAGCAATGGG-3′

### Hematoxylin and Eosin staining

The tumor, liver, lung, and bone marrow tissues were taken in the same way as above from each group of rats (*n*=10) on days 0 and 7 to monitor the morphologic change. The tissues were fixed in 4% paraformaldehyde (Shanghai Yuanye Biotechnology Co. Ltd, Shanghai, China). Hematoxylin and Eosin (H&E) staining was performed according to the common method. Briefly, the samples were dehydrated in a graded series of ethanol solutions, cleared in xylene, embedded in paraffin, cut into sections, stained with H&E, and finally observed under a microscope (BX51, Olympus, Japan).

### Scanning electron microscope

To observe microstructure, the bone marrow tissues were taken by the aforementioned method from each group of rats (*n*=10) on days 0 and 7. The tissues were first fixed with 4% glutaraldehyde (Shanghai Yuanye Biotechnology Co. Ltd, Shanghai, China) for more than 4 h, then were washed three times with the PBS, post-fixed with 1% potassium phosphate-buffered osmium tetroxide (pH = 7.0, provided by Electron Microscope Testing Lab, Chongqing Medical University, Chongqing, China) for 1 h, and washed three times with PBS. Then, the tissues were dehydrated using a graded series of ethanol (30, 50, 70, 80, 90, 95, and 100%) for 15–20 min at each step, transferred to the mixture of alcohol and isoamyl acetate (provided by Electron Microscope Testing Lab, Chongqing Medical University, Chongqing, China) for approximately 30 min, and then transferred to pure isoamyl acetate for approximately 1 h. Finally, the tissues were dehydrated in a critical point dryer (Hitachi, Japan) with liquid CO_2_. The dehydrated tissues were coated with gold-palladium (provided by Electron Microscope Testing Lab, Chongqing Medical University, Chongqing, China) and observed under a scanning electron microscope (SEM; Hitachi, Japan).

### Statistical analysis

All the data were expressed as mean ± SD. Data were statistically analyzed by the SPSS 22.0 software (IBM, U.S.A.). One-way analysis of variance and independent-samples *t* test were used to analyze the difference in groups. The Chi-square test was used to analyze the differences in the rates of diarrhea and mortality. The *P-* value less than 0.05 was considered statistically significant. The *P*-value less than 0.01 was regarded as highly statistically significant.

## Results

### Breast cancer establishment in SD rats

**Figure 1:**
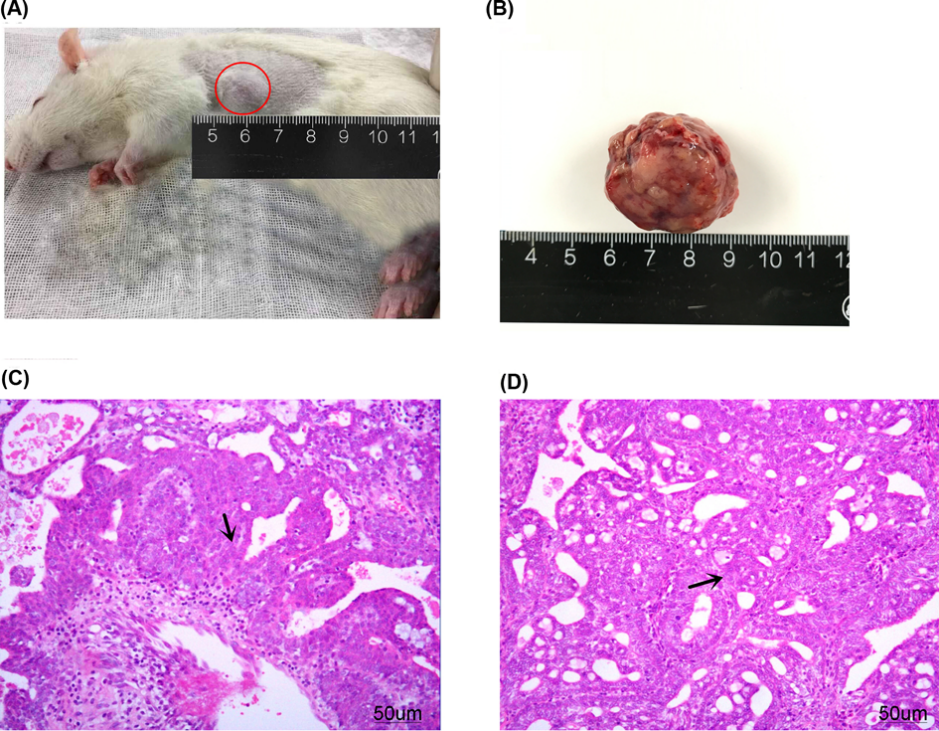
Breast cancer establishment in SD rat (**A,B**) Tumor tissue of rats with breast cancer *in vivo* and *in vitro*, respectively. (**C,D**) Breast cancer tissue was stained by H&E staining (magnification ×200). (C) Papillary adenocarcinoma, the cancer cells are papillary growth with the axis of fibrous vessels. (D) Invasive ductal carcinoma, heterocyst grows in adenoid and breaks through the basement membrane (the red circles indicate the location of the tumor, and the black arrows indicate the breast cancer cell).

After gavage with 7,12-dimethylbenz[a]anthracene, the symptoms of lack of activity, unresponsiveness, and decrease in food and water intake came under observation in rats. The incidence of breast cancer in 5–7, 8–10, 11–13, and 14–16 weeks after gavage was 0, 82, 88, and 90%, respectively. The tumor was mostly located in the first or second pairs of mammary glands (Figure 1A). The tumor tissue was removed for pathological examination (Figure 1B). According to the classification criteria of human breast cancer, the pathological types of breast cancer included papillary adenocarcinoma and invasive ductal carcinoma (Figure 1C,D). The results declared that breast cancer was established in rats successfully.

### Effectiveness of LIPUS on relieving myelosuppression

#### Diarrhea rate and mortality

**Figure 2:**
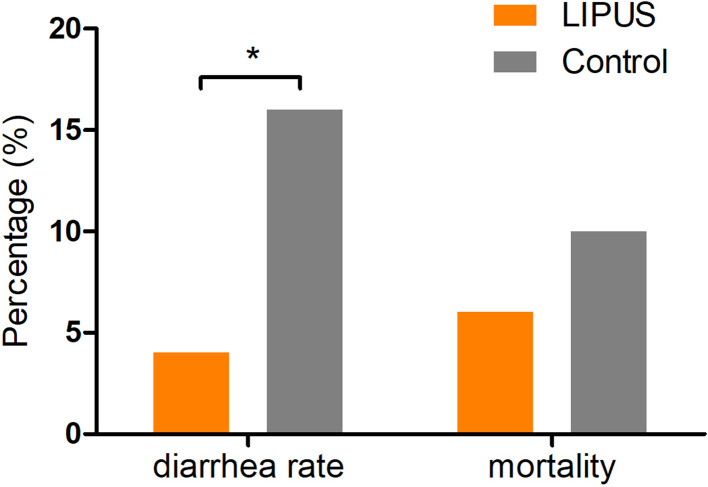
Comparison of the rates of diarrhea and mortality between LIPUS group and control group in rats with breast cancer *indicates *P*<0.05, which is statistically significant. Diarrhea rate: X^2^ = 4.000, *P*<0.05; mortality: X^2^ = 1.100, *P*>0.05.

The rats with breast cancer showed adverse reactions after CTX injection, including decrease in food and water intake, reduction in activities, lying huddled up in the cage without response, and appearance of diarrhea. The diarrhea rate in LIPUS group and control group was 4 and 16%, respectively, and there was significant difference between the two groups (*P*<0.05, Figure 2). The mortality was 6% in LIPUS group and 10% in control group and no significant difference between the two groups (Figure 2).

#### Peripheral blood cell counts

**Figure 3:**
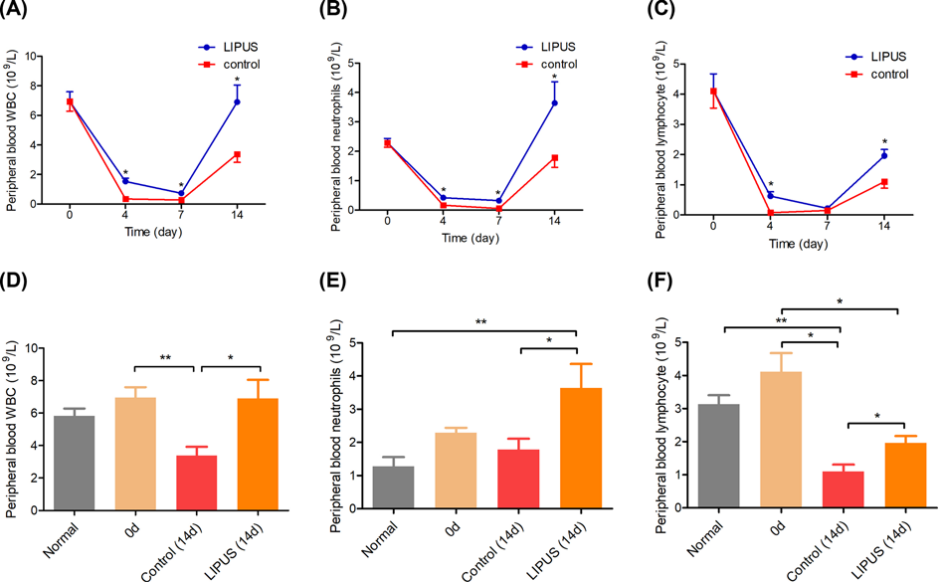
Peripheral blood cell counts (**A**) WBCs, (**B**) neutrophils, (**C**) lymphocytes in the LIPUS group and control group on days 0, 4, 7, and 14, respectively. (**D**) WBCs, (**E**) neutrophils, (**F**) lymphocytes among the normal rats, control group and LIPUS group on days 0 and 14 respectively. *P<0.05, **P<0.01.

As shown in Figure 3A, the WBC count decreased first and then increased in the LIPUS and control groups. A significant difference was found between the LIPUS and control groups on days 4, 7, and 14 (*P*<0.05). There was a difference in the number of leukocytes between the control group on days 0 and 14 (*P*<0.05, Figure 3D). The neutrophil and lymphocyte are important immune cells in the body. As shown in Figure 3B, the neutrophil count in the LIPUS group was significantly higher than that in the control group on days 4, 7, and 14 (*P*<0.05). There was a significant difference in the number of neutrophils between the LIPUS group on day 14 and normal rats (*P*<0.05, Figure 3E). The number of lymphocytes in the LIPUS group was significantly higher than that in the control group on days 4 and 14 (*P*<0.05, Figure 3C). There was a significant difference in lymphocyte count between the LIPUS group and the control group on days 0 and 14, respectively (*P*<0.05). The lymphocyte count in the normal rats was significantly higher than that in the control group on day 14 (*P*<0.05, Figure 3F).

#### Serum immunoglobulin levels

**Figure 4:**
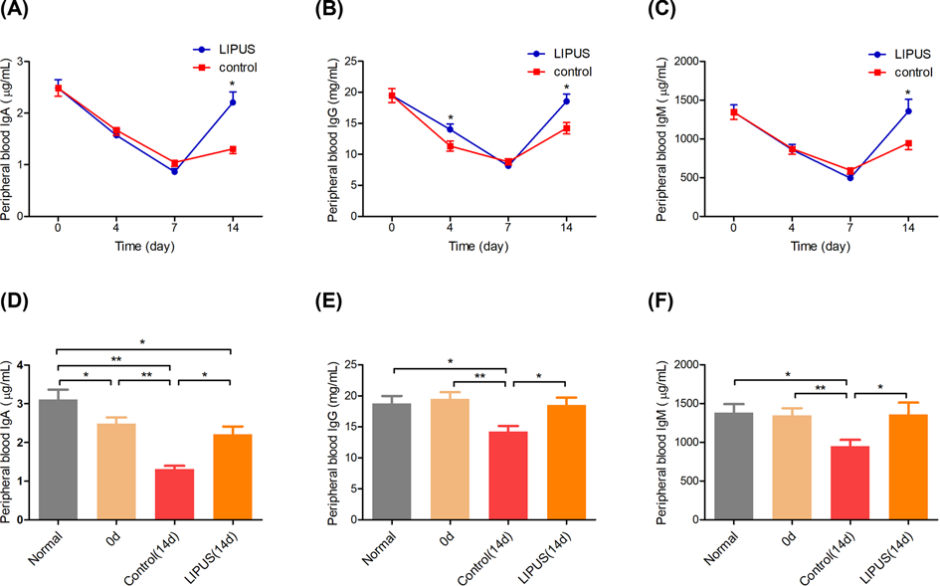
The expressions of IgA, IgG, and IgM (**A**) IgA, (**B**) IgM, (**C**) IgG in the LIPUS group and control group on days 0, 4, 7, and 14, respectively. (**D**) IgA, (**E**) IgM, (**F**) IgG among the normal rats, control group, and LIPUS group on days 0 and 14, respectively. *P<0.05, **P<0.01.

The expression of IgA, IgG, and IgM were detected to further explore the effect of LIPUS on lymphocyte secretion function. The IgA (Figure 4A), IgG (Figure 4B), and IgM (Figure 4C) decreased first and then increased in the LIPUS and control groups. The IgA and IgM in the LIPUS group were significantly higher than those in the control group on day 14 (*P*<0.05). The IgG in the LIPUS group was significantly higher than those in the control group on days 4 and 14 (*P*<0.05). There was a significant difference in IgA between the normal rats and other groups on day 0 (*P*<0.05). In the control group, IgA between days 0 and 14 was significantly different (*P*<0.05). In the LIPUS group and control group, a significant difference of IgA was shown between the normal rats and those on day 14 (*P*<0.05, Figure 4D), respectively. The IgG declared difference between the normal rats and control group on day 14 (*P*<0.05). In the control group, there was a significant difference of IgG between days 0 and 14 in (*P*<0.05, Figure 4E). The IgM was found different between the normal rats and control group on day 14 (*P*<0.05). There was a significant difference in IgM between days 0 and 14 in the control group (*P*<0.05, Figure 4F).

#### G-CSF, IL-6, and TNF-α expression

**Figure 5:**
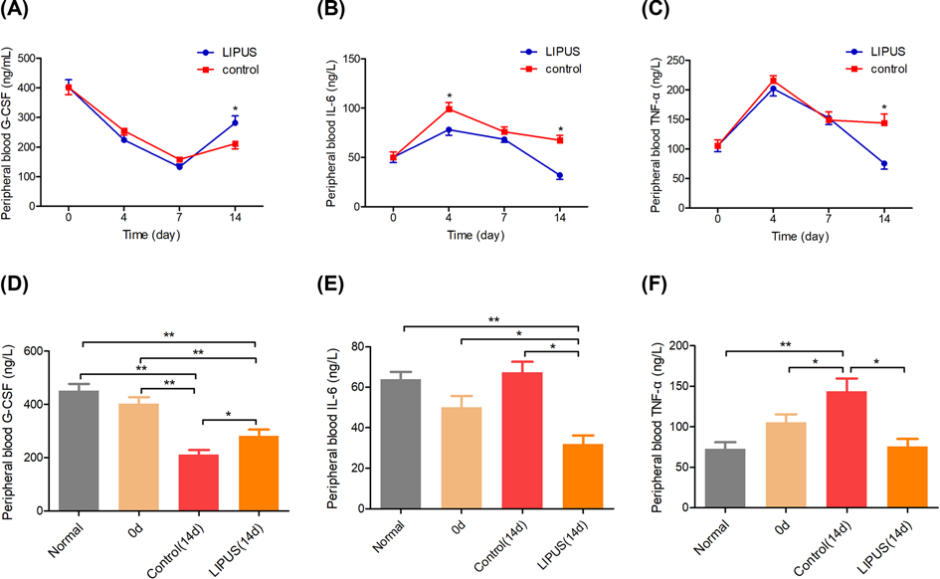
G-CSF, IL-6, and TNF-α expression (**A**) G-CSF, (**B**) IL-6, (**C**) TNF-α in the LIPUS group and control group on days 0, 4, 7, and 14, respectively. (**D**) G-CSF, (**E**) IL-6, (**F**) TNF-α among the normal rats, control group, and LIPUS group on days 0 and 14, respectively. *P<0.05, **P<0.01.

ELISA was performed to analyze the effect of LIPUS on the expression levels of G-CSF, IL-6, and TNF-α. The expression of G-CSF decreased first and then increased in the LIPUS and control groups. As shown in Figure 5A, the G-CSF was significantly higher on day 14 in the LIPUS group than in the control group (*P*<0.05). The IL-6 was significantly higher in the control group than in the LIPUS group on days 4 and 14 (*P*<0.05, Figure 5B). The TNF-α was significantly higher in the control group than in the LIPUS group on day 14 (*P*<0.05, Figure 5C). There was a significant difference in the G-CSF between the normal rats and control group and LIPUS group on day 14, respectively (*P*<0.05). There was a significant difference in the G-CSF between days 0 and 14 in control group and LIPUS group, respectively (*P*<0.05, Figure 5D). There was a significant difference of the IL-6 in normal rats and the LIPUS group on days 14 and 0 (*P*<0.05, Figure 5E). There was a significant difference between the TNF-α in normal rats and the control group on days 14 and 0 (*P*<0.05, Figure 5F).

#### Bone marrow tissues

**Figure 6:**
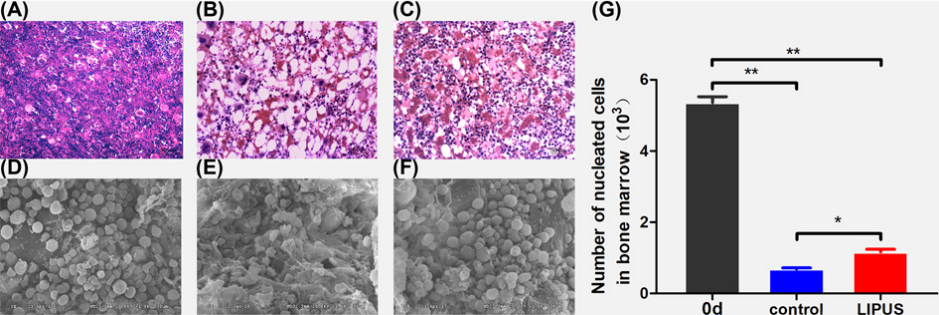
Bone marrow structure detected by SEM (magnification ×1000) and H&E staining (magnification ×200) (**A,D**) Bone marrow tissue of rats with breast cancer before CTX chemotherapy. (**B,E**) Injured bone marrow tissue of the rats after injecting CTX for 7 days. (**C,F**) Recovered bone marrow tissue from the LIPUS group after LIPUS treatment for 7 days. (**G**) Number of bone marrow nucleated cells in each group before and after treatment. *P<0.05, **P<0.01.

H&E staining and SEM were performed to analyze the effect of LIPUS on the bone marrow. The bone marrow hematopoietic tissue was rich, and cell proliferation was active in rats with breast cancer (Figure 6A,D). The structure of bone marrow in rats was destroyed on day 7, with decreased hematopoietic tissues and increased adipose tissues (Figure 6B,E). After treatment with LIPUS, the bone marrow hematopoietic tissue decreased and was different when compared with that in the control group (Figure 6C,F). The number of bone marrow nucleated cells in the LIPUS and control groups decreased on day 7 compared with that on day 0 (*P*<0.01, Figure 6G); however, the decline in the LIPUS group was lower than that in the control group (*P*<0.01).

#### Bone marrow hematopoiesis-related cytokine levels

**Figure 7:**
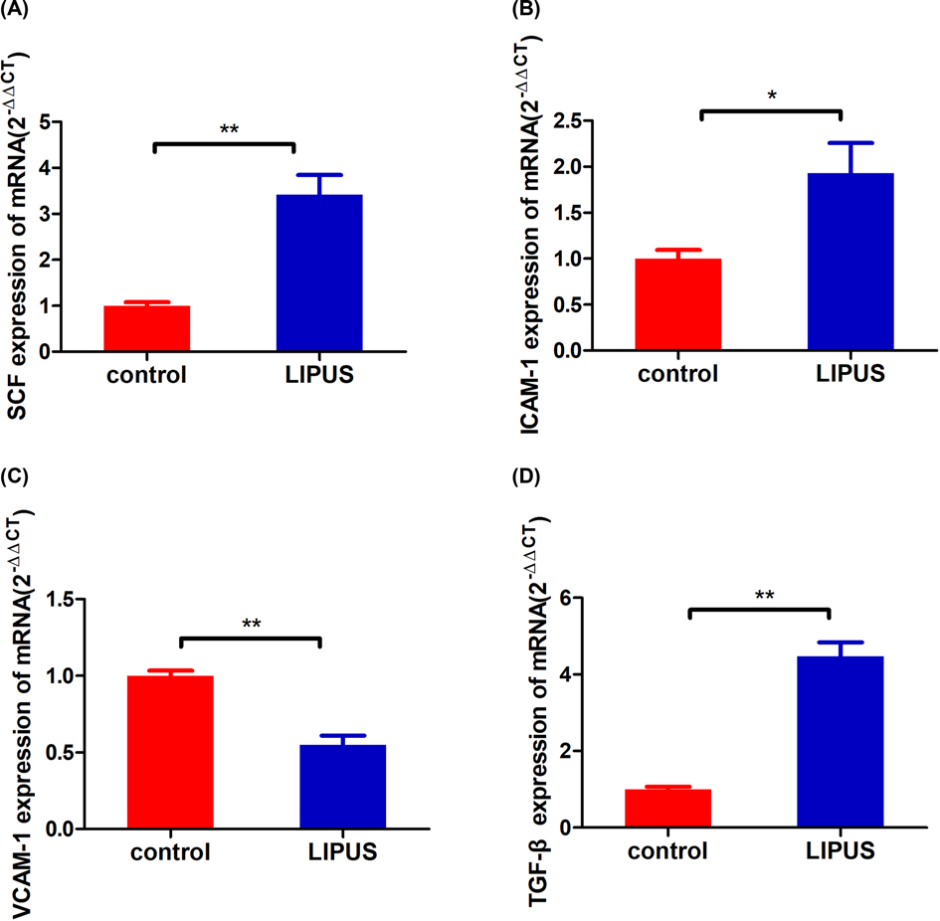
Relative mRNA expression levels (**A**) SCF, (**B**) ICAM-1, (**C**) VCAM-1, and (**D**) TGF-β in the bone marrow after injecting CTX for 7 days in the LIPUS and control groups were detected using RT-qPCR. *P<0.05, **P<0.01.

The expression of SCF, TGF-β, ICAM-1, and VCAM-1 in the bone marrow was detected to explore the hematopoietic recovery mechanism of LIPUS. The mRNA expression of SCF, ICAM-1, and TGF-β significantly increased on day 7 in the LIPUS group compared with the control group (*P*<0.05, Figure 7A,B,D). However, the mRNA of VCAM-1 was significantly higher in the control group than in the LIPUS group (*P*<0.01, Figure 7C).

### Safety of LIPUS in relieving myelosuppression

**Figure 8:**
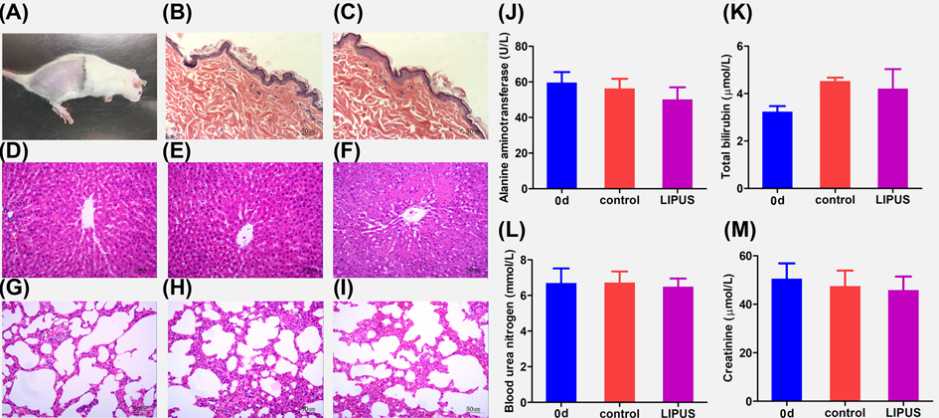
Safety of LIPUS in relieving myelosuppression (**A**) Skin of rats with breast cancer on day 0. (**B,C**) Skin of rats in the LIPUS group and control group on day 7. (**D,G**) Liver and lung tissues of rats with breast cancer on day 0. (**E,H**) Liver and lung tissues of rats in the LIPUS group on day 7. (**F,I**) Liver and lung tissues of rats in control group on day 7. The concentration of (**J**) ALT, (**K**) TBIL, (**L**) BUN, and (**M**) CRE in LIPUS group, control group on day 7, and normal rats.

The skin that received the irradiation by LIPUS was observed on day 7, which was normal without injury in LIPUS and control group (Figure 8B,C) compared with skin on day 0 (Figure 8A). There was no abnormality in HE staining of liver (Figure 8E) and lung (Figure 8H) in LIPUS group and liver (Figure 8F) and lung (Figure 8I) in control group compared with liver (Figure 8D) and lung (Figure 8G) on day 0. The concentration of ALT (Figure 8J), TBIL (Figure 8K), BUN (Figure 8L), and CRE (Figure 8M) in LIPUS group, control group and tissues on day 0 had no significant difference (*P*>0.05).

## Discussion

Chemotherapy is widely used for treating various types of tumors [25]. However, it can lead to a variety of side effects, especially bone marrow suppression [26]. Therefore, it is necessary to find a way to alleviate the chemotherapy-induced bone marrow suppression and promote the recovery of bone marrow hematopoietic function.

In previous studies, chemotherapeutic drugs were used in normal rabbits without cancer and LIPUS was used after the injection of chemotherapeutic drugs [22,23]. To be closer to clinical practice, in this study, chemotherapeutic drugs acted on rats with breast cancer and LIPUS was used with chemotherapeutic drugs at the same time.

The severe secondary infections can be triggered by leukopenia, leading to a failure of chemotherapy in clinic [27]. On day 14 of chemotherapy, leukocyte count in the control group failed to recover to the level before chemotherapy, while the leukocyte count in the LIPUS group had recovered to the level before chemotherapy. Neutrophil count in both groups recovered to the level before chemotherapy on day 14, and neutrophil count in the LIPUS group increased significantly compared with that in the normal rats. Lymphocyte count in the control group was significantly lower than that before chemotherapy and in normal rats, while lymphocyte count in LIPUS group was significantly lower than that before chemotherapy on day 14 but returned to the level in normal rats. The results revealed that LIPUS could also enhance the counts of WBCs, neutrophils and lymphocytes of rats with breast cancer after chemotherapy in the peripheral blood. Our results suggest that LIPUS treatment can reduce the severity of bone marrow suppression induced by chemotherapy when animals are undergoing cancer.

Under the stimulation of antigen, the B lymphocyte will transform into plasma cells, producing antibodies that specifically bind to the corresponding antigen, namely immunoglobulin (IG). Studies [22] have shown that LIPUS can increase the expression levels of IgA, IgM, and IgG in CTX-induced leukopenia animal model. Our research results displayed that LIPUS could effectively increase the expression levels of IgA, IgG, and IgM in the serum of rats with breast cancer after chemotherapy and enhance the immunity and anti-infection ability of the rats.

The bone marrow hematopoietic microenvironment includes bone marrow microcirculation, interstitial cells, and cytokines [28]. Chemotherapy can cause severe damage to the hematopoietic microenvironment [29]. Cytokines in the bone marrow hematopoietic microenvironment have been reported to support the growth and survival of hematopoietic stem cells [30]. The expression levels of hematopoietic cytokines were detected to explore the mechanism of LIPUS in alleviating bone marrow depression.

The SCF is the key to the survival, proliferation, and differentiation of early hematopoietic stem cells [31]. The ICAM-1 and VCAM-1 are two kinds of adhesion molecules expressed on bone marrow stromal cells. ICAM-1 mediates the adhesion and information exchange between hematopoietic and stromal cells [32], enhancing the sensitivity of hematopoietic cells to cytokines and participating in hematopoietic regulation [33]. In this study, the expression level of SCF and ICAM-1 increased significantly after the adjuvant therapy of LIPUS, which was beneficial to adhesion between bone marrow hematopoietic and stromal cells, so that bone marrow hematopoietic cells could divide, proliferate, and release, thereby improving bone marrow suppression. VCAM-1 mediates intercellular adhesion and intercellular signal transduction, which plays an irreplaceable role in the localization, proliferation, and differentiation of myeloid cells [34]. rG-CSF can stimulate the myeloid progenitor cell proliferation and promote the development and maturation of granulocytes [35]. The results showed that the expression of G-CSF increased after LIPUS treatment, G-CSF mediated the mobilization of bone marrow hematopoietic stem cells by down-regulating the expression level of VCAM-1 [36,37]. In this study, the expression level of G-CSF increased and the expression level of VCAM-1 decreased after the LIPUS treatment, which was consistent with the results of the aforementioned study. TGF-β plays a critical role in the development of CD4^+^ CD25^+^ Foxp3^+^ regulatory T (Treg) cells, as CD4^+^ T cells deficient in TGF-β signaling cannot be converted into Treg cells *in vitro* or *in viv*o [38]. Treg defects are implicated in autoimmune marrow failure [39]. In this study, the expression level of TGF-β increased after the treatment of LIPUS, which was conducive to the recovery of hematopoietic function. In addition, H&E staining and SEM were performed to analyze the effect of LIPUS on the bone marrow. The number of bone marrow nucleated cells decreased significantly after chemotherapy, LIPUS relieved myelosuppression and improved the proliferation of bone marrow.

TNF and IL-6 are known mediators of inflammation in a variety of clinical situations, and a number of therapeutic strategies have sought to block the actions of these cytokines [40,41]. The high level of TNF-α can lead to the angiogenesis of tumor, promote the growth of tumor cells, and be beneficial to the infiltration and metastasis of cancer cells [42,43]. Studies have shown that high expression levels of IL-6 and TNF-α in breast cancer can lead to a decrease in survival and a significant increase in mortality, which is beneficial to the progression and dissemination of breast cancer [44]. In this study, the level of TNF-α and IL-6 decreased significantly after the adjuvant therapy of LIPUS. This indicated that LIPUS could reduce the metastasis of tumor by decreasing the expression levels of TNF-α and IL-6.

After LIPUS treatment, skin, liver, and kidney functions were examined. There were no abnormalities in skin, liver, and kidney function after LIPUS treatment. We can speculate that ultrasound is safe in the treatment of bone marrow suppression induced by chemotherapy in breast cancer rats.

The limitations of this experiment were treatment time only following the previous report and there was no observation of subsequent effects. Many studies such as the treatment duration and survival time after treatment will be researched deeply in future.

In conclusion, the present study clearly confirmed that LIPUS could ameliorate chemotherapy-induced myelosuppression in rats with breast cancer by regulating the hematopoietic microenvironment. This provided an experimental basis for the application of LIPUS as an adjuvant therapy for treating tumors.

## Abbreviations

ALT: alanine transaminase
BUN: blood urea nitrogen
CRE: creatinine
CTX: cyclophosphamide
ELISA: enzyme-linked immunosorbent assay
G-CSF: granulocyte colony-stimulating factor
H&E: Hematoxylin and Eosin
HRP: horseradish peroxidase
ICAM-1: intercellular cell adhesion molecule-1
IL-6: interleukin-6
LIPUS: low-intensity pulsed ultrasound
PBS: phosphate buffer solution
rG-CSF: recombinant human granulocyte colony stimulating factor
RT-qPCR: reverse transcription-quantitative polymerase chain reaction
SCF: stem cell factor
SD rat: Sprague-Dawley rat
SEM: scanning electron microscope
TBIL: total bilirubin
Treg: regulatory T
VCAM-1: vascular cell adhesion molecule-1
WBC: white blood cell
